# A Conserved Ethylene Biosynthesis Enzyme Leads to Andromonoecy in Two Cucumis Species

**DOI:** 10.1371/journal.pone.0006144

**Published:** 2009-07-03

**Authors:** Adnane Boualem, Christelle Troadec, Irina Kovalski, Marie-Agnes Sari, Rafael Perl-Treves, Abdelhafid Bendahmane

**Affiliations:** 1 INRA-CNRS, UMR1165, Unité de Recherche en Génomique Végétale, Evry, France; 2 The Mina and Everard Goodman Faculty of Life Sciences, Bar-Ilan University, Ramat Gan, Israel; 3 CNRS, UMR 8601, Laboratoire de Chimie et Biochimie Pharmacologiques et Toxicologiques, Université René Descartes, Paris, France; Temasek Life Sciences Laboratory, Singapore

## Abstract

Andromonoecy is a widespread sexual system in angiosperms, characterized by plants carrying both male and bisexual flowers. Monoecy is characterized by the presence of both male and female flowers on the same plant. In cucumber, these sexual forms are controlled by the identity of the alleles at the *M* locus. In melon, we recently showed that the transition from monoecy to andromonoecy result from a mutation in 1-aminocyclopropane-1-carboxylic acid synthase (ACS) gene, *CmACS-7*. To isolate the andromonoecy gene in cucumber we used a candidate gene approach in combination with genetical and biochemical analysis. We demonstrated co-segregation of *CsACS2*, a close homolog of *CmACS-7*, with the *M* locus. Sequence analysis of *CsACS2* in cucumber accessions identified four CsACS2 isoforms, three in andromonoecious and one in monoecious lines. To determine whether the andromonoecious phenotype is due to a loss of ACS enzymatic activity, we expressed the four isoforms in *Escherichia coli* and assayed their activity *in vitro*. Like in melon, the isoforms from the andromonoecious lines showed reduced to no enzymatic activity and the isoform from the monoecious line was active. Consistent with this, the mutations leading andromonoecy were clustered in the active site of the enzyme. Based on this, we concluded that active CsACS2 enzyme leads to the development of female flowers in monoecious lines, whereas a reduction of enzymatic activity yields hermaphrodite flowers. Consistent with this, *CsACS2*, like *CmACS-7* in melon, is expressed specifically in carpel primordia of buds determined to develop carpels. Following ACS expression, inter-organ communication is likely responsible for the inhibition of stamina development. In both melon and cucumber, flower unisexuality seems to be the ancestral situation, as the majority of *Cucumis* species are monoecious. Thus, the ancestor gene of *CmACS-7*/*CsACS2* likely have controlled the stamen development before speciation of *Cucumis sativus* (cucumber) and *Cucumis melo* (melon) that have diverged over 40 My ago. The isolation of the genes for andromonoecy in *Cucumis* species provides a molecular basis for understanding how sexual systems arise and are maintained within and between species.

## Introduction

In angiosperms, sex determination results in the formation of separate male and female flowers on either the same (monoecy), or different individuals (dioecy). Several species in the Cucurbitaceae, including cucumber (*Cucumis sativus*) and melon (*Cucumis melo*), show polymorphism in their sexual systems. In these species, floral primordia are initially bisexual with sex determination occurring by the selective developmental arrest of either the male stamen or female carpel organs, resulting in unisexual flowers [1], [2]. Such variation is genetically controlled by sex determining genes that govern the developmental fate of individual flower buds, as well as the successive pattern of male, female or bisexual flowers along the shoots of the whole plant. Thus, gynoecious plants bear only pistillate flowers, androecious plants bear only staminate flowers, and monoecious plants develop a succession of male flowers, followed by female flowers. Hermaphrodites have only bisexual flowers and andromonoecious plants have both male and bisexual flowers [3], [4]. For decades, cucumber has been the model plant for the study of plant sex determination [5], [6]. In this plant, three major genes account for most sex phenotypes. *Female* (*F*) is a partially dominant gene that controls femaleness. The *F* allele causes the female phase to start much earlier and *FF* plants are gynoecious. *Androecious* (*a*) increases maleness and plants of the *aaff* genotype are androecious. The *Monoecious* (*M*) gene appears to act as a stamen suppressor in buds determined to develop a carpel [7]. The dominant allele will only allow the formation of stamen-less female flowers, as well as male flowers, while in *mm* plants, bisexual flowers form, in addition to male flowers.

The sex-determining genetic program is also affected by environmental conditions and can be manipulated by exogenous hormone treatments. Moreover, endogenous hormone levels were correlated with cucumber sexual development [3], [4]. Among the different hormones that have been implicated in cucumber sex expression, ethylene was shown to play a key-role.

In melon, sex is mainly determined by two genes, *andromonoecious* (*a*) and *gynoecious* (*g*). We recently cloned the andromonoecy gene from melon using a positional cloning and TILLING approach, and identified it as an 1-aminocyclopropane-1-carboxylic acid synthase (ACS) gene, *CmACS-7* [8]. ACS enzymes belong to a multigene family of pyridoxal 5′-phosphate (PLP)-dependent enzymes that catalyze the first committed and generally rate-limiting step in ethylene biosynthesis in higher plants [9]. We showed that *CmACS-7* is specifically expressed in carpel primordia, and, in andromonoecious genotypes, a missense mutation leads to loss of enzymatic activity. Phenotypically, melon *andromonoecious* (*a*) gene appears to act similarly to cucumber *Monoecious* (*M*) gene: in both species, the dominant allele suppresses stamen development in pistillate flowers without affecting male flower formation, whereas the recessive allele “releases” such inhibition, resulting in bisexual flowers instead of female flowers.

In the present study, we investigated whether the cucumber *Monoecious* (*M*) locus is orthologous to the melon *a* locus, and encodes for an ACS homologous to *CmACS-7*. Using a genetic approach we showed that *CsACS2*, a cucumber ACS highly homologous to *CmACS-7*, co-segregates with the *Monoecious (M)* locus. *CsACS2* like *CmACS-7* is specifically expressed in buds determined to develop a carpel in carpel primordia and, like in melon, loss-of-function mutations, located near the active site of CsACS2 are associated with andromonoecy.

## Results

### Identification of the cucumber orthologue of melon *CmACS-7*

**Figure 1:**
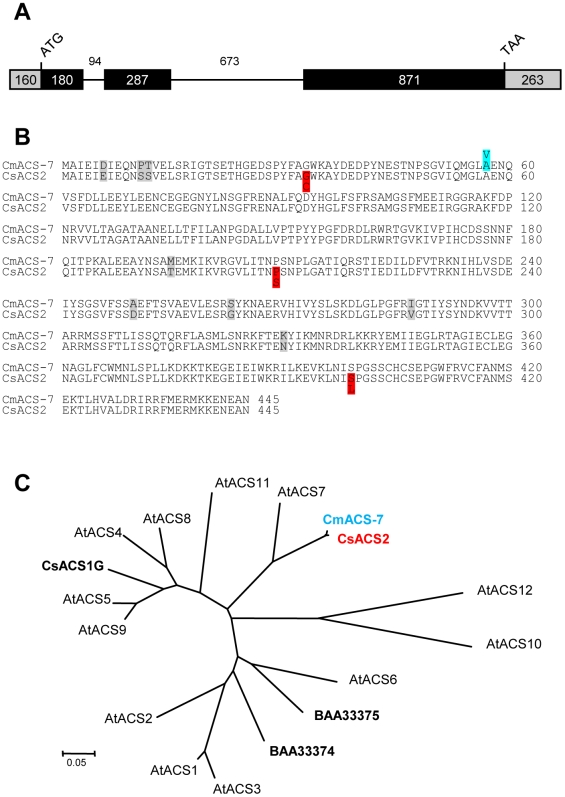
Sequence analysis of *CsACS2*. (A) Schematic diagram of *CsACS2* gene structure. The numbers indicate the size of the 3 exons (filled boxes) and the 2 introns (black lines) in bps. The grey boxes indicate the 5′ and the 3′ UTR. (B) Alignment of the melon protein CmACS-7 and cucumber CsACS2. The grey boxes indicate the 8 residues that are polymorphic between CsACS2 and CmACS-7. The blue and red boxes indicate the amino acid changes associated with the andromonoecious phenotype in melon and cucumber, respectively. (C) Relationships between CmACS-7, CsACS2 and ACS from *Arabidopsis*. CsACS1G, BAA33374 and BAA33375 are ACS from cucumber. The distance tree was produced using ClustalW to align the sequences and using a neighbour-joining algorithm to group them. The length of lines connecting the proteins indicates the mean number of estimated substitutions per site (corrected for multiple substitutions). Scale bar, 0.05 substitution per site.

In our previous study [8], we have shown that the melon *andromonoecious* (*a*) gene encodes *CmACS-7*, and that andromonoecy results from a mutation in its active site. As the cucumber *M* locus acts similarly to melon *a* gene, we asked whether the cucumber ortholog of *CmACS-7* could be encoded by the *M* locus. To test this hypothesis, we designed PCR primers to amplify the *CmACS-7* ortholog in cucumber and amplified a 2420-bp genomic fragment. Sequence analysis of the amplified DNA shows that, similar to melon, the cucumber gene contains 3 exons and 2 introns (Figure 1A), and encodes an ACC synthase of 445 amino acids that corresponds to *CsACS2* (GenBank accession no. D89732; [10]). Homology analysis showed that the coding regions of CmACS-7 and CsACS2 share 98% identity in their amino acid sequence and only differ in eight residues, all of which are located in non-conserved positions among seed plants (Figure 1B). The other three ACS family members reported in cucumber (CsACS1G and GenBank accessions no. BAA33374 and BAA33375) are more distant, and only share 53–62% amino acid identity with CmACS-7. A phylogenetic tree, representing twelve ACS sequences from Arabidopsis and cucumber show that CsACS2 and CmACS-7 are highly similar and are related to AtACS7 (Figure 1C). Thus, we conclude that *CsACS2* is likely the cucumber ortholog of *CmACS-7*.

### Co-segregation of *CsACS2* with the cucumber *M* locus

**Figure 2:**
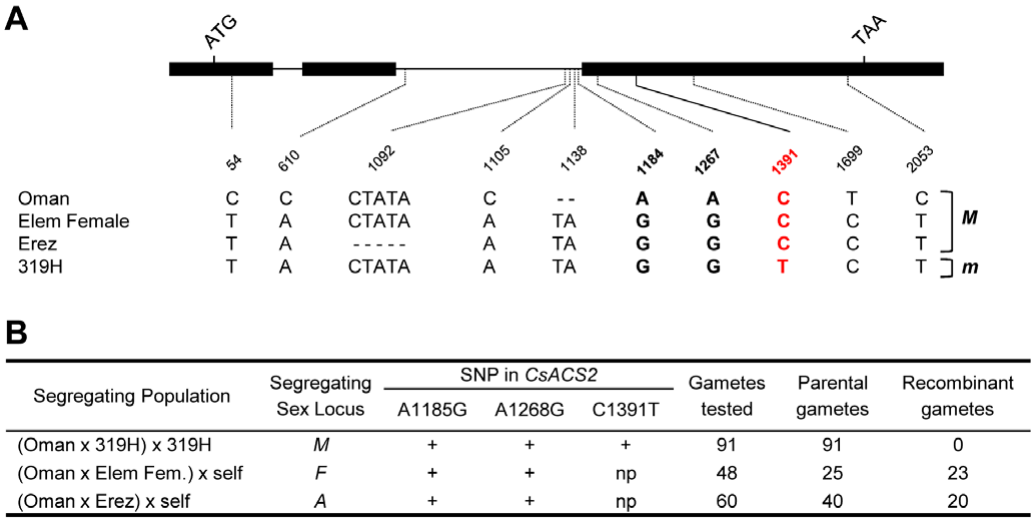
Sequence variation and co-segregation analysis. (A) *CsACS2* sequence variation in the four parental lines used in the co-segragation analysis. Polymorphic sites indicated below the gene model were positioned relative to the first nucleotide of the start codon. The red colored nucleotide indicates the C to T transition leading to the amino acid substitution P209S. Genotypes of the parental lines, Oman, 319H, Elem Female and Erez are *ffMMAA*, *FFmmAA*, *FFMMAA* and *ffMMaa*, respectively. *M* and *m* genotypes on the left indicate whether the accessions harbour the dominant *M* or the recessive *m* alleles at the *M* locus. (B) Linkage analysis of *Female* (*F*), *androecious* (*a*) and M*onoecious* (*M*) loci with *CsACS2*. SNP positions are indicated relative to the first nucleotide of the start codon of the genomic *CsACS2* sequence and are shown in bold in the panel A. At position 1184 and 1267, Oman cultivar carries an A whereas 319H, Elem Female and Erez cultivars carry a G nucleotide. At position 1391, Oman, Erez and Elem Female carry a C, whereas 319H carries a T. “+” and “np” indicate polymorphic and non polymorphic nucleotide position in the plant crosses, respectively. The number of gametes carrying parental combinations of *CsACS2* haplotype and the sex genotype, and the number of gametes exhibiting non parental combinations (“recombinant” gametes) are indicated.

To map *CsACS2* relative to the sex loci *F*, *A* and *M*, we first sequenced the *CsACS2* gene in the four cucumber parental lines, Oman (*MMffAA*), Erez (*MMffaa*), Elem Female (*MMFFAA*) and 319H (*mmFFAA*), used to generate the mapping populations (Figure 2B). Among the four accessions, we observed 5 DNA polymorphisms in the second intron and 5 in the exons (Figure 2A). Of the 5 polymorphisms in the coding region, four were silent and only the SNP at nucleotide position 1391 produced a proline to serine amino acid substitution at position 209 in the protein (P209S; Figure 1B, 2A). The identified SNPs were mapped relative to *F*, *A* and *M* sex loci (Figure 2B). In this analysis the *F* and *A* loci segregated independently of *CsACS2*, based on the high proportion of “recombinant” gametes exhibiting a non-parental combination of sex genotype and *CsACS2* haplotype (23 recombinant gametes out of 48, and 20 out of 60, for the *F* and the *A* loci, respectively; Figure 2B). In contrast, a perfect co-segregation of *CsACS2* with the *M* gene was obtained in a segregating population of 91 backcross plants (Figure 2B, Table S1). Based on the functional similarity between monoecy versus andromonoecy in melon and cucumber, the high sequence identity between *CmACS-7 and CsACS2* and the co-segregation of the *M* gene and *CsACS2* we concluded that it is likely that the *M* gene encodes for *CsACS2*.

### Structural and biochemical basis for *CsACS2* allelic variations

**Figure 3:**
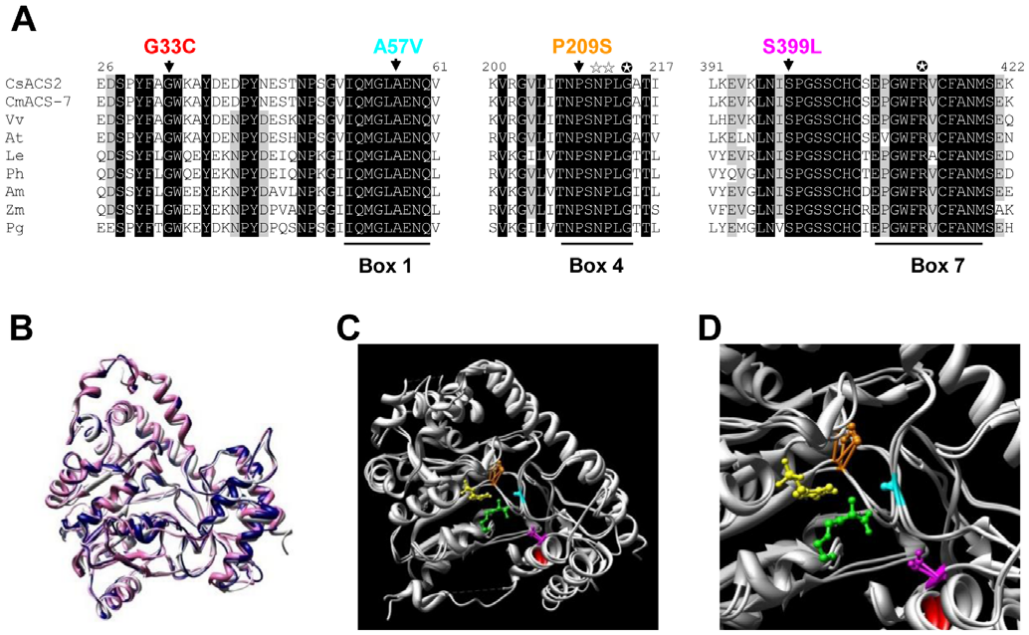
Amino acid mutations associated with andromonoecy are located at the active site of CsACS2. (A) Amino acid alignments of CsACS2, CmACS-7 and homologous proteins from *Vitis vinifera* (*Vv*), *Arabidopsis thaliana* (*At*), *Lycopersicon esculentum* (*Le*), *Petunia hybrida* (*Ph*), *Antirrhinum majus* (*Am*), *Zea mays* (*Zm*), *Picea glauca* (*Pg*). Numbers above the alignment indicate the amino acid positions along the CsACS2 protein. Box 1, Box 4 and Box 7 indicate conserved domains in ACS. The conserved residues between ACS and subgroup 1 aminotransferases and the invariant residue in all aminotransferases are indicated by ⋆ and 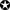, respectively. Melon A57V and cucumber G33C, P209S and S399L sequence variations are shown above the alignment (B–D) 3D structure model of CsACS2. (B) Superposition of the tomato ACS structure determined by x-ray crystallography [13], indicated in white and the 3D models of CsACS2, indicated in blue, and of CmACS-7, indicated in pink. The melon and cucumber models were determined using the Geno3D server (http://geno3d-pbil.ibcp.fr). (C and D) Zoom in of the ACS active site. Ball and stick representations show the cofactor PLP in yellow, the competitive inhibitor AVG in green, and the three amino acids G^33^ in red, P^209^ in orange and S^399^ in magenta associated with the presence of hermaphrodite flowers in cucumber. The amino acid A^57^, previously shown to be associated with andromonoecy in melon, is represented in cyan [8].

To understand the basis of allelic differences in the *M* gene, we compared the sequence of *CsACS2* in 28 cucumber accessions (Table 1). In the absence of structured cucumber core collections, accessions were selected based on their sexual types and geographic origin to cover the maximum genetic diversity. All tested monoecious, gynoecious and androecious accessions harbouring the dominant *M* allele presented the same CsACS2 protein sequence. In contrast, hermaphrodite and andromonoecious accessions, harbouring the recessive *m* allele, revealed three protein isoforms, G33C, P209S and S399L, each differ by a single amino acid mutation of CsACS2 (Figure 1B). When the closest ACS homologous sequences from different plants were aligned, we observed that the residues G^33^, P^209^ and S^399^ are conserved across seed plants (Figure 3A; [11]). P^209^ and S^399^ residues are located in the conserved box 4 and 7, respectively, in close proximity to invariant residues that are conserved in all aminotransferases (Figure 3A; [12]). Crystallographic studies have determined that residues in the box 4 and 7 are involved in binding of the PLP cofactor and the enzyme substrate *S*-adenosyl methionine (SAM), respectively. The G^33^ residue is located nearby the residues P^29^, Y^30^ and F^31^ that contribute to the hydrophobic pocket where adenine ring of SAM is positioned (Figure 3B–D; [13]).

**Table 1:** Cucumber germplasms used in this study.

Accession	Source	Sex type	Genotype	Amino acid 33	Amino acid 209	Amino acid 399	Bisexual flowers
Erez	Zeraim Gedera	androecious	*MM ff aa*	G	P	S	no
PI 369717	Poland	androecious	*MM ff aa*	G	P	S	no
249-2	Hazera Genetics	gynoecious	*MM FF AA*	G	P	S	no
Elem Female	Zeraim Gedera	gynoecious	*MM FF AA*	G	P	S	no
Beth Alfa	Zeraim Gedera	monoecious	*MM ff AA*	G	P	S	no
CGN22246	Czechoslovakia	monoecious	*MM ff AA*	G	P	S	no
ECD-mono.	Hannover Univ.	monoecious	*MM ff AA*	G	P	S	no
ED-mono.	Hannover Univ.	monoecious	*MM ff AA*	G	P	S	no
Market More 76	Toronto Univ.	monoecious	*MM ff AA*	G	P	S	no
Oman	Kew Gardens UK	monoecious	*MM ff AA*	G	P	S	no
Poinsett 76	Indam Seeds	monoecious	*MM ff AA*	G	P	S	no
Shimshon	Zeraim Gedera	monoecious	*MM ff AA*	G	P	S	no
Wrd-mono.	Hannover Univ.	monoecious	*MM ff AA*	G	P	S	no
Ames 3950	Australia	andromonoecious	*mm ff AA*	**C**	P	S	**yes**
CGN21634	Nigeria	andromonoecious	*mm ff AA*	**C**	P	S	**yes**
CGN22937	Australia	andromonoecious	*mm ff AA*	**C**	P	S	**yes**
PI 292012	Israel	andromonoecious	*mm ff AA*	**C**	P	S	**yes**
PI 618932	China	andromonoecious	*mm ff AA*	**C**	P	S	**yes**
SGN22933	USA	andromonoecious	*mm ff AA*	**C**	P	S	**yes**
319H	Michigan State Univ.	hermaphrodite	*mm FF AA*	G	**S**	S	**yes**
CGN22938	URSS	hermaphrodite	*mm FF AA*	G	**S**	S	**yes**
ECD-hermaph.	Hannover Univ.	hermaphrodite	*mm FF AA*	G	**S**	S	**yes**
ED-hermaph.	Hannover Univ.	hermaphrodite	*mm FF AA*	G	**S**	S	**yes**
PI 351139	Former soviet union	hermaphrodite	*mm FF AA*	G	**S**	S	**yes**
PI 356809	Former soviet union	hermaphrodite	*mm FF AA*	G	**S**	S	**yes**
PI 370643	Former soviet union	hermaphrodite	*mm FF AA*	G	**S**	S	**yes**
WrD-hermaph.	Hannover Univ.	hermaphrodite	*mm FF AA*	G	**S**	S	**yes**
CGN 22961	Former soviet union	hermaphrodite	*mm FF AA*	G	P	**L**	**yes**

**Figure 4:**
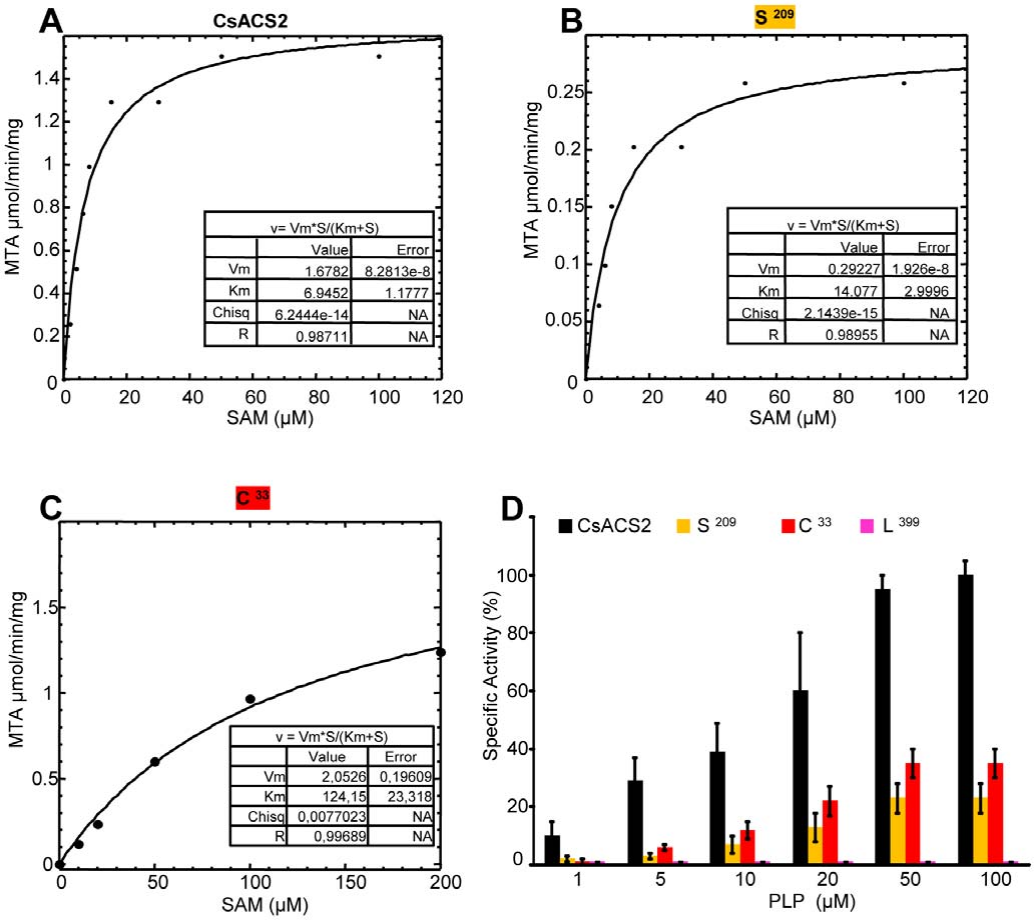
Biochemical characterization of S^209^, C^33^ and L^399^ CsACS2 isoforms. (A–C) Initial velocity measured at 50 µM of PLP. Experimental data (circle) were fit into a line following Michaelis-Menten equation. The resulting parameters are indicated in the table (insert). (D) Enzymatic activity of CsACS2 (black bars), S^209^ (orange bars), C^33^ (red bars) and L^399^ (magenta bars) protein forms. Specific activities were measured on dialyzed enzymes in the presence of 60 µM SAM and various PLP concentrations.

To determine whether hermaphrodite flowers in andromonoecious and hermaphrodite plants is due to loss of a CsACS2 enzymatic activity, we expressed the four different forms of the protein as six-histidine tag (His_6_-CsACS2) fusion proteins in *Escherichia coli*. The purified proteins were assessed for enzymatic activity *in vitro* by monitoring 5′-methylthioadenosine (MTA) formation at different PLP concentrations. The L^399^ isoform was found to be totally inactive. The C^33^ isoform has a reduced activity and no tendency towards saturation was observed up to 200 µM of SAM leading to a 15-fold increase in the *Km* value compared to CsACS2 isoform (Figure 4, Table 2). The S^209^ ACS isoform has a reduced enzymatic activity; its *V_max_* is approximately 17% of the *V_max_* of the CsACS2 isoform (Figure 4, Table 2). Consistent with this, in tomato, a P^209^ ACS mutants generated by site-directed mutagenesis displayed more than 95% reduction of the enzymatic activity [14]. P^209^ is a conserved residue of ACS box 4 involved in PLP binding. The decrease in the enzyme activity is likely due to less efficient binding of the PLP cofactor [13]. Taking all these data together and because the three isoforms from the lines homozygote for the *m* alleles showed reduced to no enzymatic activity and the unique isoform from the lines harbouring the dominant *M* alleles was active we concluded that the loss of CsACS2 enzyme activity is likely the cause of the apparition of hermaphrodite flowers in cucumber.

**Table 2:** The kinetic parameters, Km and Vm, measured for CsACS2, S^209^ and C^33^ protein isoforms.

Enzyme	Km (µM)	Vm (µmol.min^−1^.mg^−1^)
CsACS2	7±1.5	1.7±0.1
S^209^	13±3	0.3±0.02
C^33^	104±3	2.0±0.3

L^399^ isoform was not included in this table as it is completely inactive.

### *CsACS2* is expressed in carpel primordia of female and hermaphrodite flowers

**Figure 5:**
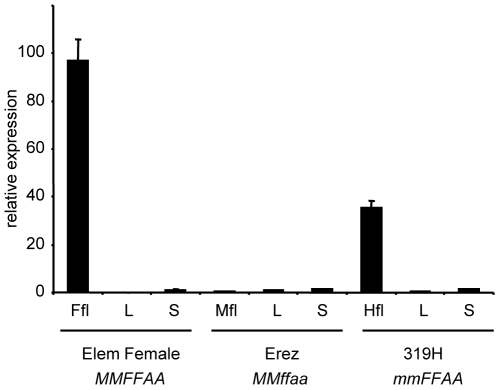
Expression analysis of *CsACS2*. Expression of *CsACS2* in different organs was determined using quantitative real-time PCR, relative to *CsACTIN2* standard. The mean±s.d. of four biological replicate experiments is shown. L, Leaf; S, Stem; Mfl, Male flower; Hfl, Hermaphrodite flower and Ffl, Female flower. Cucumber accessions and their respective genotypes are indicated below the graph.

In cucumber, a detailed morphological description divided the flower meristem development into 12 distinct stages before anthesis [15]. The critical stage at which development of the inappropriate sexual organ is arrested occurs at stage 6, just after the elaboration of carpel primordia [15]. *In situ* expression analysis carried out by Saito et al. [16] showed that in gynoecious and monoecious cucumber accessions, *CsACS2* mRNA began to accumulate during stage 4 and its expression continues at later stages. The early accumulation of *CsACS2* mRNA was localised in the carpel primordia of flower determined to develop as female and not detected in male flower primordia of the monoecious plant. Since the *in situ* hybridizations by Saito et al. [16] involved monoecious and gynoecious accessions harbouring the *M* allele, we investigated *CsACS2* expression in a cucumber hermaphrodite accession bearing the *m* allele. Our quantitative RT-PCR expression analysis demonstrates that *CsACS2* mRNA is mainly expressed in female and hermaphrodite flowers (Figure 5). The lower transcript levels that we observed in the bisexual flowers could be explained by the response of *CsACS2* expression to ethylene [17], resulting in a positive feedback effect on gene expression; the ACS *m* allele produces less ethylene and is therefore expressed at lower levels. *CsACS2* mRNA was not detected in male flowers at any developmental stage (Figure 5), similar to the observations of Saito et al. [16]. Altogether, these results reveal that *CsACS2* and the melon *andromonoecious* (*CmACS-7*) gene have the same expression pattern [8].

The *CsACS2* transcripts are expressed in both female and hermaphrodite flowers (Figure 5; [16]), but the loss of CsACS2 protein activity accounts for the functional variation between the *M* and *m* alleles (Figure 4). We therefore concluded that CsACS2-mediated ethylene production in the carpel primordia likely prevents the development of the stamina in female flowers, but is not required for carpel development, since both *mm* and *MM* plants develop a functional carpel.

## Discussion

In melon and cucumber, flower unisexuality seems to be the ancestral situation, as the majority of the *Cucumis* species are monoecious [18]. Based on our previous studies and the present work, we have demonstrated that the ancestor gene of *CmACS-7*/*CsACS2* performed the stamen suppressing activity before speciation of *Cucumis sativus* and *Cucumis melo*. These two species, differing in chromosome number and probable geographic origin, belong to two different clades in the Genus *Cucumis* and were estimated to have diverged over 40 My ago [19], [20]. Variants with pistillate-bisexual flowers appeared much later: the point mutations that reduced ACS activity are different in the two species. Interestingly, the same gene has been independently selected, in both clades, to “engineer” a bisexual flower. Andromonoecy is a major sexual system that has evolved independently numerous times [21] and is found in ∼4,000 species in 33 angiosperm families [22]. It has been suggested that andromonoecy is selected to permit flexibility in resource allocation to male and female function, and is thus found in species where fruit set is resource-limited [21], [23], [24], or for elevating male function, to increase pollen donation [25].

Identifying *M* locus as an ethylene biosynthesis gene expressed in the carpel is rather surprising. Models that predicted the action of *F* and *M* suggested that *F* determines threshold levels of ethylene that are required to perform a dual action: promote pistil development and inhibit stamen formation in the pistillate flowers [4], [7], [26]. The action of *F* appears to be gradual and “systemic”: femaleness increases along the shoot and can “diffuse” from an *FF* rootstock to an *ff* scion [27]. The action of *M* appears more local and does not diffuse across grafts [28]. The above models suggests that *M* encodes a protein involved in sensing inhibitory ethylene in the stamens, *mm* plants being insensitive to such inhibition. Indeed, Yamasaki et al. [7] showed that *mm* plants were less responsive to ethylene; the assays included inhibition of hypocotyl elongation, of stamen elongation, and reduced induction of several ethylene responsive transcripts by ethephone. In fact, several ethylene biosynthesis and sensing genes such as *ACS, ACO, ERS1 and ETR2* respond to ethylene by increasing their transcript levels [17]. Therefore, lack of ethylene production at a critical site could prevent the synthesis of receptors, rendering the particular tissue ethylene-insensitive. It remains, however, unclear why exogenous supply of ethylene to *mm* plants does not cure their deficiency [7]; possibly, the developmental window for the receptors to respond or ACC to act [29] is very narrow. The application of ethylene or its precursor on the whole plant could have a pleiotropic effect that masks the direct effect of ethylene on *m*-mediated sexual function.

The expression patterns of *CsACS2* were shown to correlate with femaleness: *CsACS2* transcripts increased when monoecious plants entered the female phase [7], [10], [30], [31], or were exposed to short photoperiods that enhance femaleness [31]. Spatial expression patterns of *CsACS2* were reported by Yamasaki et al. [30] and by Saito et al. [16] to be closely related to the fate of specific flower buds. *CsACS2* mRNA was strongly localised in the carpel primordia of flower determined to develop as female and not detected in male flower primordia. By quantitative RT-PCR we showed that *CsACS2* is mainly expressed in female and hermaphrodite flowers. These data are similar to those we recently reported in melon for *CmACS-7* [8].

The observation that the ethylene signal required to inhibit the stamens is generated in a different tissue, the carpel primordium, and must be translocated to the target tissue, is puzzling. Ethylene has a dual role and is also required at the same time for carpel development; applying exogenous hormone, or engineering melons to over-express ACS, resulted in increased femaleness [32]. Nevertheless, melon and cucumber plants expressing inactive *CsACS2* isoforms are not compromised in carpel formation, suggesting that other ACS isoforms provide the carpel-promoting function. Thus, *CsACS2* is dedicated to stamen arrest, whereas other ACS isozymes that act in the carpel, such as the *F*-locus that encodes *CsACS1G*, cannot affect the stamens [33]–[35]. Such specificity of enzymes that produce a gaseous end-product could seem counter-intuitive. It implies that ethylene synthesis and perception are regulated in spatially restricted patterns that change during development and among sex types. The pistil-located ACS could export ACC towards the stamen, where ethylene will eventually form by a local ACC oxidase. The other ACS isoforms present in the carpel could be expressed at different stages or tissues, such that ACC export does not occur at the critical time. This could ensure that the inhibitory signal is only activated in pistillate flowers that express *CsACS2*, and not in male flowers. This provides a molecular explanation to the differential action of *M* on male versus female flowers.

The intricacy of inter-organ signaling during sex determination became apparent in a study by Little et al. [36], who blocked ethylene sensing in specific floral whorls by over-expressing a deficient *ETR1* allele. Contrary to the author's predictions, blocking ethylene perception in the stamen primordia prevented carpel formation. This suggests an unexpected role for ethylene perception in the stamens that serves not only to repress stamen development, but also to promote carpel development. In our case, ACC formation in carpels is spatially separated from ethylene perception in the stamens.

## Methods

### Plant material and segregating populations

Cucumber genotypes of different sex types used for this study are listed in Table 1. To perform linkage analysis of sex determining genes, three segregating populations were prepared. A common monoecious accession, *Cucumis sativus var. hardwickii* from Dhofar, Oman (“Oman”, accession 0092760, Kew Botanical Gardens, UK) was crossed with cultivated cucumber lines to maximize the level of polymorphism between the partners and facilitate mapping. For analyzing the segregation of the *Female (F)* gene, a gynoecious line, Elem Female (*FFMMAA*) was crossed with the monoecious genotype, ‘Oman’ (*ffMMAA*), the F_1_ was self-fertilized and an F_2_ population was produced. For the *androecious* (*a*) gene segregation, ‘Oman’ was crossed with an androecious line, Erez (*ffMMaa*), and the F_1_ was selfed to produce the F_2_ population. To analyze the segregation of the *Monoecious (M)* gene, ‘Oman’ was crossed with an hermaphrodite line 319H, genotype *FFmmAA*, and the F_1_ hybrid was back-crossed to 319H to produce a BC_1_ progeny. The rest of the genotypes in Table 1 served for the accession comparison of the *CsACS2* sequences.

Plants were grown in the greenhouse in the spring and summer in 10 L pots under standard agronomic conditions and evaluated for the sex of their flowers at each node along the main stem and side branches 2–3 times, for at least 30 nodes of the main stem. Individual plants' leaves were sampled and DNA was extracted according to Baudracco-Arnas [37].

### Plant genotyping

To identify plants carrying recombination events, plant DNA was extracted from each individual of the three segregating populations. Determination of the sexual phenotype was performed for all plants. For genotyping, a 302 pb DNA fragment of the *CsACS2* gene was amplified with primers listed in Table S2 and sequenced. Polymorphisms within this fragment were used to determine recombinant alleles.

### Quantitative RT-PCR and RACE-PCR experiments

Total RNA was extracted from frozen leaves, stems and flowers using the Trizol reagent (Invitrogen). To avoid sampling contamination and to accurately assay the *CsACS2* mRNA expression, we used cucumber accessions bearing only one flower type: the female, male and bisexual flower buds were collected from the gynoecious line Elem Female, hermaphrodite line 319H, and from the androecious line, Erez, respectively. Contaminating DNA was removed by DNaseI treatment (Invitrogen). First-strand cDNA was synthesized from 2 µg of total RNA using the Superscript® III reverse transcriptase (Invitrogen). Primer design was performed using the Primer3 software (http://frodo.wi.mit.edu/cgi-bin/primer3/primer3_www.cgi). Primers sequences used are listed in Table S2. To check the specificity of the designed primers, all amplicons were sequenced and blasted against NCBI database. Polymerase chain reactions were performed in an optical 384-well plate with an ABI PRISM® 7900 HT Sequence Detection System (Applied Biosystems) apparatus, using qPCR MasterMix Plus for SYBR® Green I w/o UNG (Eurogentec) and according to manufacturer's instructions. Cycling conditions were as follows: 50°C for 2 min; 95°C for 10 min; 40 cycles of 95°C for 15 sec and 60°C for 1 min. PCR amplification specificity was verified by a dissociation curve (55°C to 95°C). A negative control without cDNA, technical replicates on three independent cDNA samples (derived from the same RNA sample), and three independent biological experiments were performed in all cases. Data were analysed using the SDS 2.0 software (Applied Biosystems). To compare data from different PCR runs and cDNA samples, C_T_ values for *CsACS2* were normalized to the C_T_ value of *CsACTIN2* (primers shown in Table S2). *CsACS2* relative expression was determined as described in Czechowski et al. [38].

### Bacterial strains, plasmids and chemicals

The BL21 (DE3) pLYSS *E. coli* strain {*F^−^ ompT hsdS_B_(r_B_^−^ m r_B_) gal dcm (DE3) pLysS (Cm^R^)*} was used for enzyme protein expression. The plasmid pET15b (Novagen) expesses the insert under the T7 promoter, and confers ampicillin resistance. S-Adenosyl Methionine (SAM), Pyridoxal 5′phosphate (PLP) and 5′Adenylic Acid Deaminase from Aspergillus required for ACS activity assays were purchased from Sigma.

### Expression of recombinant CsACS2 in E. coli

BL21 (DE3) pLYS cells transformed with CsACS2 protein expression constructs were incubated in 25 ml of Luria-Bertani medium (tryptone 10 g/L, yeast extract 5 g/L, NaCl 10 g/L) supplemented with ampicillin and chloramphenicol (50 µg/ml each) and incubated overnight at 37°C. This pre-culture was used to inoculate 2 L of the same medium supplemented with ampicillin (50 µg/ml), and the cells were grown at 37°C in a shaking incubator at a speed of 180 rpm until OD_600_ = 0,6. IPTG was added (0.5 mM ) to induce protein expression and cells were grown for 5 hours at 25°C, harvested by centrifugation and kept overnight at −45°C. The frozen cells were resuspended in 15 ml of 50 mM TRIS pH 7.9 and 500 mM NaCl buffer, disrupted on ice by sonication in the presence of the protease inhibitors phenylmethanesulfonyl fluoride, leupeptin, pepstatin, and aprotinin (10 µg/ml each). The cell debris were removed by centrifugation at 13000 g for 15 min and the supernatant was immediately used for enzyme purification.

### Purification of recombinant CsACS2

The supernatant was applied to a Ni-IDA 15 ml column (Sigma) equilibrated with 50 mM Tris at pH 8, 500 mM NaCl buffer. The column was washed with 50 mM Tris at pH 8, 500 mM NaCl buffer supplemented with 10 mM imidazole until no protein was detected in the flow-through. Wild type or mutant forms of CsACS2 were eluted using the same buffer supplemented with 100 mM imidazole and dialyzed against 50 mM K-Phosphate buffer at pH 8.5 in the absence of PLP, and then concentrated using a Millipore Amicon® Ultra device (5,000 MWC0). The concentrated fraction (20 mg/ml) of CsACS2 was aliquoted and stored at −45°C in the presence of 25% glycerol until use. Protein purification was performed by capillary electrophoresis on an Experion® device (Bio Rad), using PRO260 chips, according to the manufacturer's instructions.

### Partial purification of Adenosine Deaminase

*Aspergillus* adenosine deaminase purchased from Sigma was further purified by ethanol fractionation according to Wolfenden et al. [39]. Specifically, 5 g of lyophilized deaminase powder were suspended in 90 ml of cold water in a glass beaker and 47 ml of acetone were added. The mixture was stirred at 4°C for 5 min then centrifuged at 2000 g for 1 min. The resulting pellet was mixed with 33 ml of water and stirred for 5 min, centrifuged at 2000 g for 5 min and the pellet was discarded. Ten ml of ethanol were added to the supernatant, the mixture was stirred at 4°C for 5 min and centrifuged again. Twenty ml of Ethanol were added to the supernatant and gently stirred at 4°C for 3 hours. The alcoholic mixture was centrifuged for 5 min at 7000 g and the pellet resuspended with 6 ml of water. The protein solution was then dialyzed against a 5 mM solution of sodium acetate at pH 5.3 for at least 24 hours. The resulting solution was concentrated using a Millipore Amicon® Ultra device (5,000 MWC0) in the presence of glycerol (20%) to 5 mg/ml of protein, aliquoted and stored at −45°C until use. The ethanol precipitated deaminase was routinely found to be 1000 times more active than the original powder (activity measured in 100 mM HEPES, pH 5.5 buffer using 5′AMP as a substrate, data not shown).

### ACS activity assays

ACC synthase activity was determined by monitoring MTA formation by differential spectroscopy, recorded on an Uvikon 940 spectrophotometer (Biotek-Kontron Instruments) according to White et al. [40]. Specific activity measurements were performed in triplicate using 3 different enzyme preparations. We incubated 60 µM SAM in 100 mM K-Phosphate buffer, pH 8.5 (0.2 ml), and adenosine deaminase (8 µg for 0.2 ml) in the absence or presence of PLP (ranging in concentration from 0 to 100 µM) in a quartz cuvette for 3 min at 25°C, after addition of the purified enzyme (1 to 2 µg). The conversion of the MTA produced by ACS into an inosine derivative was monitored at 265 nm (**Δ**ε = −7740 M^−1^.cm^−1^) and specific activity was expressed as mol of MTA formed per min per mg of protein. The same protocol was followed for Vm and Km determination with a concentration of SAM ranging from 1 to 100 µM.

### Protein structure modeling

The CsACS2 three-dimensional structures were generated using the Geno3D server (http://geno3d-pbil.ibcp.fr). Superposition of the tomato ACS structure (1IAY.pdb) determined by x-ray crystallography [13], and our three models of CsACS2, was carried out and visualized using the Chimera server (http://www.cgl.ucsf.edu/chimera).

### Phylogenetic analysis

Multiple sequence alignment of full-length protein sequences was performed using the ClustalW (http://www.ebi.ac.uk/Tools/clustalw2). Phylogenetic trees were constructed by MEGA4 (http://www.megasoftware.net/index.html) based on the Neighbor-Joining method.

## Supporting Information

Table S1 Segregation analysis of andromonoecious (m) locus with CsACS2. Detailed scores for the parental lines, and 91 back-cross-1 individual progeny, are displayed for the sex phenotype (Mm, monoecious, mm, hermaphrodite) and for 3 SNPs in the CsACS2 genomic sequence. SNP C1391T causes the amino acid P209S substitution that causes the allelic M/m difference.
(0.06 MB XLS)

Table S2 Primers used in this study
(0.03 MB XLS)
